# Constitutive Differential Features of Type 2 Transglutaminase in Cells Derived from Celiac Patients and from Healthy Subjects

**DOI:** 10.3390/ijms21041231

**Published:** 2020-02-12

**Authors:** Gaetana Paolella, Merlin Nanayakkara, Silvia Sposito, Marilena Lepretti, Salvatore Auricchio, Carla Esposito, Maria Vittoria Barone, Stefania Martucciello, Ivana Caputo

**Affiliations:** 1 Department of Chemistry and Biology, University of Salerno, 84084 Fisciano, Italy; gpaolella@unisa.it (G.P.); ssposito@unisa.it (S.S.); mlepretti@unisa.it (M.L.); cesposito@unisa.it (C.E.); smartucciello@unisa.it (S.M.); 2 Department of Translational Medical Science, University Federico II, 80138 Naples, Italy; merlinnanayakkara@yahoo.it (M.N.); mv.barone@unina.it (M.V.B.); 3 European Laboratory for the Investigation of Food-Induced Diseases (ELFID), University Federico II, 80138 Naples, Italy; salauric@unina.it

**Keywords:** type 2 transglutaminase, celiac disease, gliadin peptide 31–43, celiac cellular phenotype, skin-derived fibroblasts

## Abstract

Type 2 transglutaminase (TG2) is a ubiquitous enzyme able to modify gliadin peptides introduced into the organism through the diet. By means of its catalytic activity, TG2 seems to have an important pathogenetic role in celiac disease (CD), an inflammatory intestinal disease caused by the ingestion of gluten-containing cereals. A strong autoimmune response to TG2 characterizes CD development. Anti-TG2 antibodies specifically derange the uptake of the α-gliadin peptide 31–43 by control, but not by celiac dermal fibroblasts, underlying some different constitutive features regarding TG2 in healthy and celiac subjects. Our aim was to investigate whether these differences depended on a different TG2 subcellular distribution and whether peptide 31–43 differentially regulated TG2 expression and activity in cells of the two groups of subjects. We found that TG2 was more abundantly associated with membranes of celiac fibroblasts than of control cells, in particular with the early endosomal and autophagic compartments. We also found that peptide 31–43 differentially affected TG2 expression and activity in the two groups of cells, activating TG2 more in control than in celiac cells and inducing TG2 expression in celiac cells, but not in control ones. The different TG2 subcellular localization and the different way the peptide 31–43 modulates TG2 activity and availability into control and CD cells suggested that TG2 is involved in the definition of a constitutive CD cellular phenotype, thus having an important and still undefined role in CD pathogenesis.

## 1. Introduction

Type 2 transglutaminase (TG2) is a ubiquitous multifunctional protein belonging to a family of cross-linking enzymes widely distributed in animals, plants, and microorganisms [1]. The main catalytic activity of TG2 consists of the Ca^2+^-dependent formation of an isopeptide bound between the γ-carboxamide group of a glutamine residue and the ε-amino group of a lysine residue, both belonging to the same protein or to different proteins. Polyamines can also be used in this reaction. In the absence of available amines, TG2 can deamidate a glutamine residue to form glutamic acid. Moreover, TG2 may act as GTPase, disulfide-isomerase, kinase, and isopeptidase in different cell compartments and biological contexts [2]. Finally, TG2 may exert signaling/scaffolding/adapter functions, which are independent of its enzymatic activities, both inside and outside the cell [3]. All these enzymatic and non-enzymatic functions are finely tuned not only by TG2 localization into the cell (nucleus, cytosol, mitochondria, membrane inner face, cell surface, extracellular environment), but also by the availability of enzymatic regulators (mainly Ca^2+^ and GTP) and of substrates, as well as by the interaction with other protein partners [2,3,4,5]. As a consequence, TG2 takes part in many biological processes linked to cell survival, differentiation, death, and response to several kinds of stresses [3,6]. At the same time, given the crucial regulatory function of TG2 in cell life and death, it plays a key role in several pathologic conditions (cancer, neurodegenerative disorders, fibrosis, etc.) [7,8,9].

Interestingly, TG2 is the target of a strong autoimmune response in celiac disease (CD), an intestinal inflammatory disorder caused by the ingestion, in genetically predisposed individuals, of wheat gliadin and related prolamins in other cereals (secalin in rye and hordein in barley) [10,11,12]. The genetic susceptibility to CD is mainly due to the presence of particular haplotypes (DQ2 and DQ8) of human leukocytes antigens (HLA). Gliadin presentation by these molecules leads to the recruitment and activation of gliadin-specific CD4+ lymphocytes, which, in turn, trigger a strong intestinal inflammation [11]. Consequently, the intestinal mucosa becomes hyperplastic and atrophic with severe negative consequences for absorption and barrier functions [11]. TG2 seems to exacerbate the immune response to gliadin. Indeed, TG2-catalyzed deamidation of specific glutamines introduces some negative charges into gliadin peptides. Consequently, gliadin recognition by HLA-DQ2/DQ8 is more efficient, and, as a result, gliadin becomes more immunogenic [13,14]. In addition, the cross-linking function of TG2 leads to the formation of covalent complexes between gliadin and TG2 itself [10]. These complexes are processed by TG2-specific B cells, which, in turn, are stimulated by gliadin-specific T cells to produce antibodies to TG2 [15]. These autoantibodies display several biological effects by interacting with TG2 present in the extracellular environment or on the cell surface [16,17,18,19,20]. Therefore, it has been proposed that anti-TG2 antibodies have an active role in disease onset and progression [21]. A particular ability of anti-TG2 antibodies is relative to their functional interaction with the α-gliadin peptide 31–43 (p31–43). This peptide is not immunogenic and is not deamidated by TG2, but it is able to initiate both a stress and an innate immune response in CD [22]. In a previous work, we found that anti-TG2 antibodies interfered with the uptake of p31-43 by Caco-2 cells, a model of human intestinal epithelial cells, whereas they did not influence the uptake of the immunogenic α-gliadin peptide 57–68 (p57–68) [23]. The same effect was observed in skin-derived fibroblasts of healthy subjects. Surprisingly, anti-TG2 antibodies influenced the uptake of p31-43 by skin-derived fibroblasts of CD patients little [24].

In the present study, we aimed to establish how TG2 may contribute to the different way p31-43 is internalized by celiac and control dermal fibroblasts. Thus, we investigated whether cells from control subjects and CD patients showed differences in TG2 localization in different cellular compartments. In the attempt to identify other constitutive differences regarding TG2 in control and celiac cells, we also investigated whether p31–43 differentially modulated TG2 expression and activity into fibroblasts of the two groups of subjects.

## 2. Results

### 2.1. Subcellular Distribution of TG2

**Figure 1:**
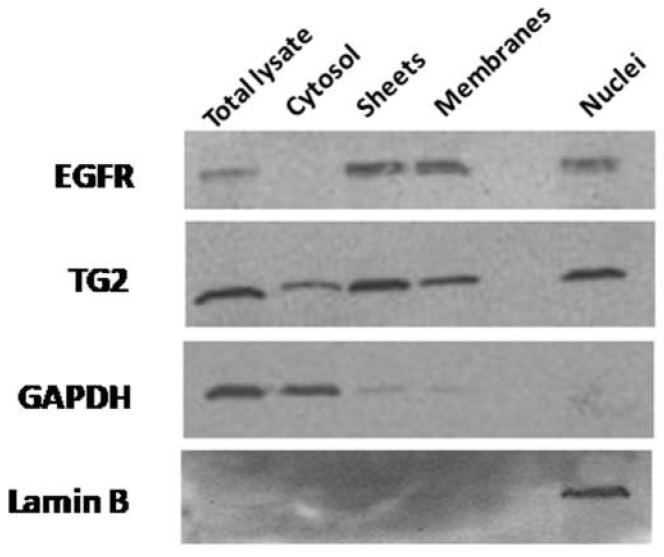
Subcellular fractionation of TG2 in primary fibroblasts. Western blot analysis showing TG2 levels in subcellular fractions of one representative sample (from a CD culture). The identity of each fraction was verified by analyzing the expression of specific markers, such as the EGF-receptor (EGFR) for membrane fractions (sheets and membranes), GAPDH for the cytosol, and lamin B for the nuclear fraction.

**Figure 2:**
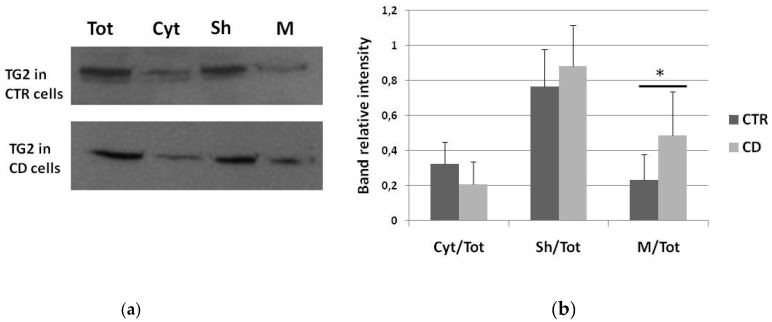
TG2 subcellular distribution in cytosolic and membrane fractions. (**a**) Western blot anti-TG2 on total lysates and on cytosolic and membrane fractions of fibroblasts from one representative control subject and one representative celiac subject (Tot, total lysate; Cyt, cytosol; Sh, sheets; M, membrane). (**b**) Graphical representation of mean values (and standard deviations) of densitometric analyses of relative TG2 levels into each fraction, expressed as the ratio between band intensity of the fraction and band intensity of the respective total lysate. The graph is relative to Western blots performed on four control samples and four CD samples. Asterisk (*) indicates that *p* is < 0.05.

We performed fractionating experiments to identify differences relative to TG2 distribution into different cell compartments. We first verified the efficacy of the fractionating protocol on a CD sample (Figure 1), then we focused our attention on how TG2 is relatively more abundant in cytosol or membrane fractions of four control and four celiac samples. Western blot analysis showed that the amount of cytosolic TG2 was slightly lower in CD cells than in controls, whereas TG2 associated with membrane sheets did not vary between the two groups (Figure 2). On the contrary, the portion of TG2 associated with the membrane fraction was slightly, but significantly more abundant in celiac fibroblasts with respect to control ones (Figure 2).

### 2.2. TG2 on the Membrane Surface

**Figure 3:**
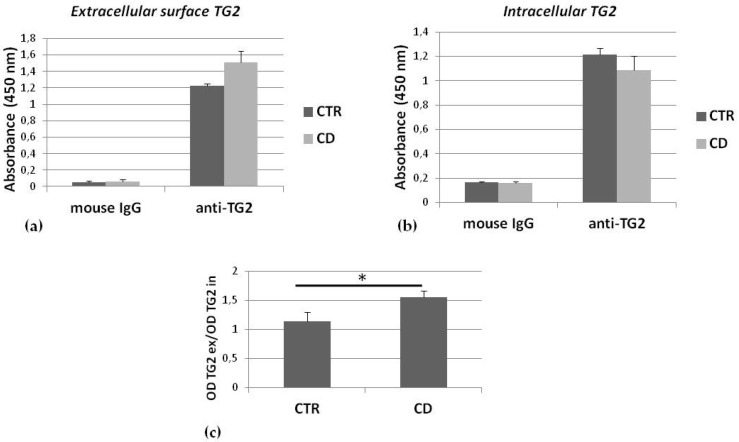
Relative amount of TG2 associated with the extracellular membrane surface. Absorbances relative to detection of TG2 on cell surface (**a**) and of intracellular TG2 (**b**) of one representative control sample and one representative CD sample. In both (**a**) and (**b**), absorbances relative to non-specific mouse IgG, used as negative control, are also shown. Each determination is made in triplicate. (**c**) Graphical representation of mean values (and standard deviations) of ratios between absorbance (OD) relative to surface TG2 (TG2_ex_) and to intracellular TG2 (TG2_in_), measured in corresponding wells, referred to analysis performed on three control and three CD cultures. Asterisk (*) indicates that *p* is < 0.05.

To evaluate the portion of membrane TG2 associated with the cell surface, we measured, by a microplate immune assay, the relative amount of TG2 associated with the membrane surface of living cells. Comparing one control sample and one CD sample, we found that the absorbance relative to extracellular surface TG2 was higher for the CD culture than the control one (Figure 3a), while absorbance relative to intracellular TG2 was slightly lower for the CD culture than the control one (Figure 3b). The ratio between absorbance relative to surface TG2 and to intracellular TG2, measured in corresponding wells, for three control and three CD cultures indicated that there was a slight, but significantly higher association of TG2 with the surface cell membrane in CD cells than in controls (Figure 3c).

### 2.3. Intracellular Colocalization of TG2 with Vesicular Markers

**Figure 4:**
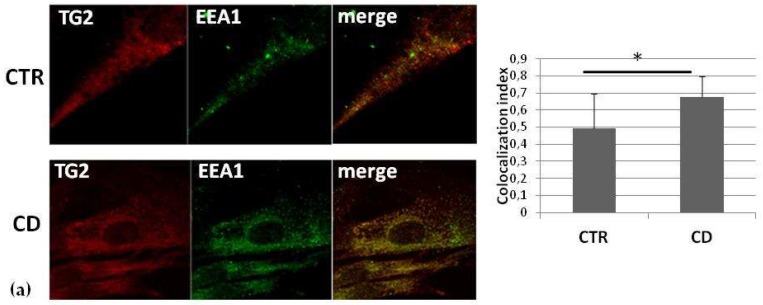

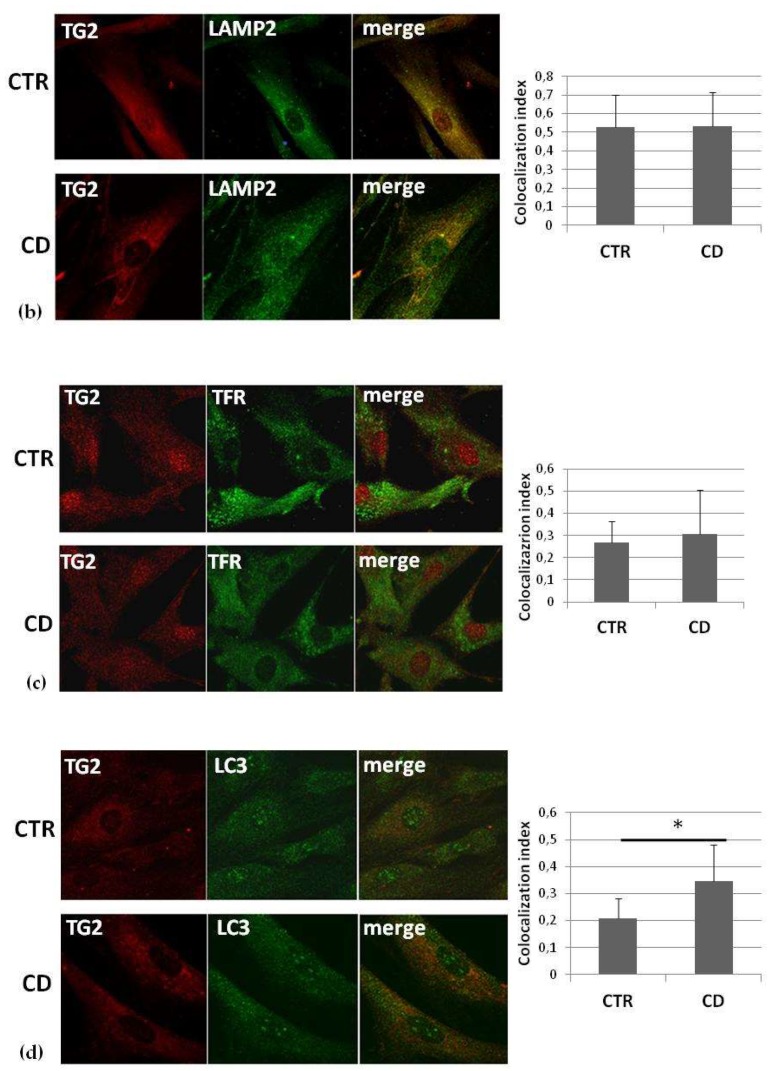
TG2 colocalization with endosomal markers. Confocal immunofluorescence images (magnification 63×) of fibroblasts from control and CD subjects stained with antibodies against TG2 (red) and EEA1 (green) (**a**)**,** LAMP2 (green) (**b**), transferrin receptor (TFR) (green) (**c**), and LC3 (**d**); the merging of red and green fields is shown in yellow. Graphs resume colocalization data regarding experiments on four control and four CD samples. Asterisk (*) indicates that *p* is < 0.05.

We investigated TG2 colocalization with markers of different intracellular membrane compartments in an attempt to establish whether there were constitutive differences in TG2 distribution between CD and control cells. Confocal microscopic images revealed that TG2 colocalized with the early endosome antigen 1 (EEA1), a marker of the early endosomal compartment, in both groups of cells, but we found a higher colocalization between TG2 and EEA1 in CD fibroblasts than in control cells (Figure 4a). TG2 also colocalized with the lysosome-associated membrane protein 2 (LAMP2), a marker of the late endosomal compartment, in both groups of cells, without any difference between the two groups (Figure 4b). TG2 colocalization with the transferrin receptor, a marker of recycling vesicles, did not reveal significant differences between the two groups (Figure 4c). Finally, TG2 colocalization with the microtubule-associated protein1A/1B-light chain 3 (LC3), a marker of the autophagic compartment, showed a higher colocalization between TG2 and LC3 in CD cells than in control ones (Figure 4d).

### 2.4. Effects of p31–43 on TG2 Activity and Expression

**Figure 5:**
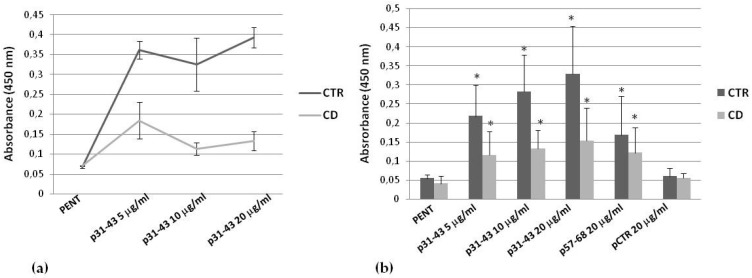
Effect of p31–43 on in situ TG2 activity. (**a**) Comparison of TG2 activity induced by 5, 10, and 20 μg/mL of p31–43 in one representative control culture and one representative CD culture. Basal activity of TG2 was obtained in the presence of pentylamine-biotin (PENT) only. Means and standard deviations of biological duplicates are reported. (**b**) Mean values (and standard deviations) relative to in situ TG2 activity measured in three control cultures and three CD cultures treated with 5, 10, and 20 μg/mL of p31–43 and with 20 μg/mL of control peptides. Asterisks (*) indicate that *p* is < 0.05 vs. respective pentylamine-biotin-treated cells.

To demonstrate whether p31-43 was able to modulate intracellular TG2 activity in skin-derived fibroblasts, we performed an in situ enzymatic assay. First, we compared activity measured in one culture from a healthy subject and one culture from a CD subject, chosen for their high level of TG2 expression (not shown). We observed that p31–43 induced an increase in TG2 activity; however, TG2 activation was clearly less pronounced in the celiac sample (Figure 5a). Mean values relative to TG2 activity in three control samples and three CD samples, expressing similar levels of TG2 (not shown), confirmed a dose-dependent increase of TG2 activity in the presence of p31–43, but again showed a generally lower activation in celiac cells than in control ones (Figure 5b). We also observed a significant TG2 activation in the presence of p57–68 (20 μg/mL) (Figure 5b). In these experiments, we used the A-gliadin peptide 229–246 (p229–246) at 20 μg/mL as the irrelevant control peptide (Figure 5b).

**Figure 6:**
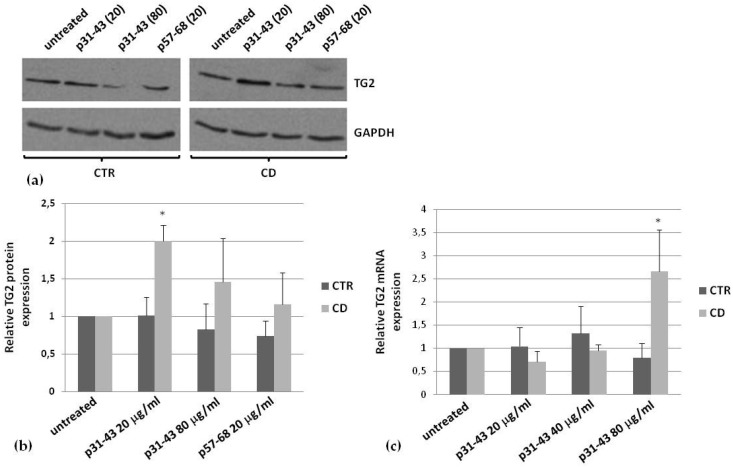
Effect of p31–43 on TG2 expression. (**a**) Representative western blot of TG2 expression induced by a treatment for 48 h in the presence of p31–43 (20 and 80 μg/mL) and p57–68 (80 μg/mL). (**b**) Densitometric analysis of blots relative to three control and three CD samples. In all experiments, 35 μg of total proteins were loaded for analysis. GAPDH was used as the internal reference. (**c**) Real-time PCR analysis to quantify relative amounts of TG2 in the presence of 20, 40, and 80 μg/mL of p31–43, in three control and three CD samples. Asterisks (*) indicate that *p* < 0.05 vs. respective untreated samples.

Then, we monitored TG2 expression in the presence of p31–43. In cells from control cultures, p31–43 did not induce TG2 expression after 48 h of treatment, even at a high peptide concentration (80 μg/mL), whereas, in CD cells, we observed a significant increase of TG2 protein level already at 20 μg/mL (Figure 6a,b). As control peptide, we used p57–68, which only slightly induced TG2 expression in CD cells (Figure 6a,b). To verify whether the increase in TG2 protein was related to an increase of mRNA production, we performed a real-time PCR analysis. We found that TG2 mRNA was over-expressed (about 2.5 fold), in CD cells only, in the presence of p31–43 80 μg/mL and not at lower concentrations (Figure 6c).

## 3. Discussion

Molecular bases of gliadin toxicity in CD subjects is strictly related to the deamidation of specific glutamines of immunogenic peptides, catalyzed by TG2 at the level of the intestinal mucosa [25]. Gut lumen is also reached by another class of gliadin peptides, of which p31–43 is the prototype, able to trigger a local innate immune and proliferative response [22]. p31–43 enters cells, probably by a direct interaction with cell membranes [26], and induces a wide range of biological effects, such as induction of a Ca^2+^ signaling, actin rearrangement, proliferation, endocytic delay, etc. [22]. Antibodies to TG2, abundantly produced at a very early stage of the disease [21], specifically derange p31–43 uptake by non-celiac cells [23,24], suggesting a general role for surface TG2 in regulating p31–43 endocytosis, even if neither TG2, nor another cell surface protein seem to be involved in p31–43 uptake [27]. However, anti-TG2 antibodies are inefficient at reducing p31–43 uptake by celiac cells [24], thus failing in their hypothetical protective function towards the negative effects of p31–43.

The different way employed by celiac and control cells to internalize p31–43 well fits with the concept that celiac cells, independently of their type and localization in the body, display constitutive alterations with respect to non-celiac cells [28,29]. For example, a generally higher level of phosphorylation and an increased number of focal adhesions were observed in celiac cells, such as dermal fibroblasts, far from the main site of inflammation [29]. Moreover, a constitutive alteration of early to late vesicular trafficking has been observed in CD enterocytes and dermal fibroblasts, with an increase of stress and inflammation markers, which could render CD cells more sensitive to the effects of gliadin peptides [30]. Altogether, these observations recently led to the definition of a “celiac cellular phenotype”, i.e., a condition independent of gliadin exposure and also evident far from the intestine [28].

In this paper, we report findings that confirm and reinforce the idea that TG2 localization and function into CD cells contribute to the celiac cellular phenotype (Table 1). We investigated how TG2 distributed into different compartments of control and celiac cells, specifically primary skin-derived fibroblasts, a useful model to study constitutive differences. Our biochemical data indicated that, in CD fibroblasts, TG2 was more associated with the membrane fraction and that it was slightly more abundant on the cell surface than in controls. It is well established that TG2 associates with the endosomal compartment and recycling vesicles [31], as well as with autophagosomes upon autophagy induction [32]. Confocal microscopy analyses on fibroblasts, investigating TG2 colocalization with the main markers of the endosomal compartment, demonstrated that TG2 was associated with EEA1-positive vesicles (but not with LAMP2-positive vesicles) more in celiac than in control cells. Thus, in CD cells, TG2 seems to persist into the early endosomal compartment, and consequently, its targeting to the lysosomal compartment could be delayed. On the contrary, we did not observe differences in colocalization with the transferrin receptor, indicating that TG2 was associated with the recycling compartment in a similar way in control and CD cells. Finally, TG2 appeared to colocalize with the autophagic marker LC3 more in CD cells than in control ones. This finding could be related to the complex role of TG2 in regulating autophagy [33,34], a process that could contribute, if deranged, to CD pathogenesis [35,36].

It has been demonstrated that p31–43 is able to induce TG2 activity and expression in Caco-2 cells [37]. Thus, we investigated the relationship between exposure of skin-derived fibroblasts to p31–43 and TG2 expression and activity. We observed relevant differences between CD and control cells. Indeed, p31–43 rapidly induced TG2 intracellular activation, but in CD cells, this activation was less pronounced. In a previous work, we excluded that TG2 possessed different catalytic properties in CD cells with respect to control cells, as the TG2 of both groups were activated by ionomycin and inhibited by a specific inhibitor [24]. Thus, the lower activation of TG2 in CD cells could be due to the presence, in CD cells, of a TG2 inhibitor or of a competitor of TG2 substrates. Another explanation could be related to a different regulation of intracellular Ca^2+^ homeostasis, particularly regarding the endoplasmic reticulum or mitochondria, which are the target of the Ca^2+^ mobilizing-activity of p31–43, as demonstrated in Caco-2 cells [37]. In addition, when we analyzed TG2 expression after a prolonged treatment (48 h) with p31–43, we found an increased level of TG2 protein in CD cells only, whereas no significant modification of TG2 expression could be observed in control cells. However, we found an increase of TG2 mRNA in CD cells, only at the highest concentration of p31–43, suggesting that the increase of the TG2 protein could be due, at least in part, to a modulation of TG2 turnover. The ability of p31–43 to delay endosomal trafficking [28] could also reduce the rate of TG2 endocytosis and consequent degradation into lysosomes. On the whole, p31–43 appeared to modulate TG2 availability into CD cells with unknown consequences for cell homeostasis.

### Conclusions

In conclusion, TG2 is associated with the membrane fraction, in particular the cell surface, the early endosomal compartment, and the autophagic compartment, more in CD fibroblasts than in control cells. Moreover, p31–43 differently regulates TG2 activity and enzyme availability in CD and control cells. On these bases, we could affirm that TG2 contributes to defining the celiac cellular phenotype. The biological consequence of this contribution needs further in-depth investigations. However, we can speculate that in the environment of the intestinal CD mucosa, a slightly higher amount of TG2, potentially active, on the cell surface, could favor gliadin cross-linking and deamidation, thus enhancing the immune and auto-immune response. In addition, a higher amount of TG2 in the early endosomal compartment could enhance gliadin deamidation at slightly acidic pH. Finally, p31–43 could enhance TG2 level into CD cells, thus leading to a vicious circle that contributes to triggering the immune response in CD mucosa.

## 4. Materials and Methods

### 4.1. Primary Fibroblasts Culture

Fibroblasts were obtained from skin biopsies of five CD patients on a gluten-free diet (age range 17–43 years) and five HLA-DQ2-negative healthy controls (age range 25–30 years). Celiac patients were on a gluten-free diet for at least 4 years and showed normal biopsies (Marsh T_0_) and negative serology (anti-TG2 antibody levels ranging between 0 and 1.6 U/mL); they also were negative for anti-endomysium antibodies. None of the CD patients were affected by dermatitis herpetiformis. Fibroblasts were cultured in Dulbecco’s Modified Eagle’s Medium supplemented with 20% (*v/v*) fetal bovine serum, 1 mM L-glutamine, 50 U/mL penicillin, and 50 μg/mL streptomycin (Invitrogen SRL, Milan, Italy). Cells were maintained at 37 °C in a 5% CO_2_, 95% air-humidified atmosphere. In all experiments, fibroblasts were used between passages 4 and 7.

### 4.2. Peptides

Peptides p31–43 and p57–68, i.e., LGQQQPFPPQQPY and QLQPFPQPQLPY, respectively, were synthesized on the sequence of the α-gliadin (Inbios, Naples, Italy). As the irrelevant control peptide, a synthetic peptide, representing the C-terminal sequence of A-gliadin (p229–246), was used. Each peptide was solubilized into serum-free medium at 10 mg/mL and stored in small aliquots at −20 °C.

### 4.3. Subcellular Fractionation

**Figure 7:**
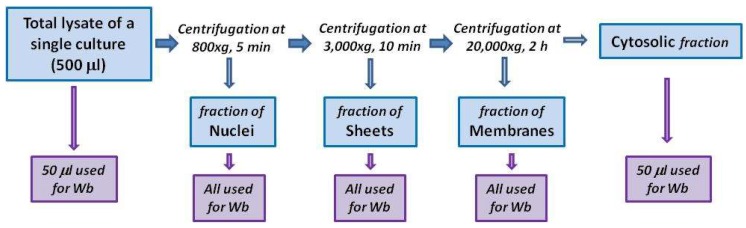
Scheme of the fractionating protocol, starting from 500 μL of total lysate; pellets, representing nuclear, sheet, and membrane fractions, were recovered and loaded onto an SDS-page for Western blot (Wb) analysis. Western blot was also conducted on aliquots (50 μL) of cytosol and total lysate.

To monitor TG2 distribution in cytosol and membranes, cells were mechanically harvested in hypotonic lysis buffer, consisting of 10 mM Tris-HCl, pH 7.5, 1 mM EDTA, 1 mM Na_3_OV_4_, 1 mM PMSF, and an inhibitor cocktail of proteases (Sigma-Aldrich, Milan, Italy). Then, cells were disrupted by passing them through a 25 Ga needle several times using a 1 mL syringe. After 30 min of incubation on ice, 500 μL of total lysate were centrifuged at 800× *g* for 3 min at 4 °C to remove nuclei and unbroken cells. The obtained supernatant was centrifuged at 3000× *g* for 10 min at 4 °C to obtain a sheet membrane fraction [38]. The supernatant was then centrifuged at 20,000× *g* for 2 h at 4 °C to obtain the membrane fraction. The sheet fraction and the membrane fraction were each resuspended in 50 μL of Laemmli sample buffer. For Western blot analyses, 50 μL of total lysate, of cytosolic fraction, and of both sheet and membrane fractions were loaded into the gel (scheme of the protocol in Figure 7). Protein content was determined by using the Bio-Rad reagent protein (Bio-Rad Laboratories, Milan, Italy).

### 4.4. Detection of Membrane-Surface TG2

To obtain a relative estimation of TG2 amount on the cell surface of skin-derived fibroblasts, cells were seeded at 8000/cm^2^ in wells of a 96 well microplate and cultured for 24 h. Then, cells were incubated for 30 min at 4 °C with mouse anti-TG2 antibodies (clone CUB 7402), 1:50 in 5% bovine albumin in phosphate-buffered saline (PBS) with Ca^2+^ and Mg^2+^. Then, cells were fixed for 5 min with 3% paraformaldehyde and incubated for 30 min with anti-mouse horseradish peroxidase-conjugated antibodies, diluted 1:100. In parallel wells, cells were first fixed and permeabilized (with 0.2% Triton-X 100 for 5 min), then incubated with 1:100 CUB 7402 and with secondary antibodies, as described above. At the end of incubation, the horseradish peroxidase substrate 3,3′,5,5′-tetramethylbenzidine (Sigma-Aldrich,) was added to each well, and absorbances were measured at 450 nm after 30 min. Finally, we calculated the ratio between absorbances relative to TG2 on the cell surface (i.e., when the primary antibody was used without fixation) and absorbances relative to intracellular TG2 (i.e., when the primary antibodies were used after fixation and permeabilization). In these experiments, we used non-specific mouse IgG (Santa Cruz Biotechnology Inc., Santa Cruz, CA, USA) as the control of the unspecific staining. Furthermore, we stained all wells with the crystal violet dye (Sigma-Aldrich) to confirm that the same amount of cells was present in each well (adapted from Feoktistova et al. [39]).

### 4.5. Confocal Microscopy

Fibroblasts were cultured on glass cover slips for 48 h, then were fixed with 3% paraformaldehyde for 10 min and permeabilized with 0.2% Triton-X 100 for 5 min. Cells were incubated for 1 h with primary antibodies diluted in 1% bovine albumin in PBS as follows: mouse anti-TG2 (CUB 7402), 1:150, rabbit anti-TG2, 1:280, and mouse anti-transferrin receptor, 1:500 (Thermo Fisher Scientific, Monza, Italy); mouse anti-LAMP2, 1:100, and goat anti-EEA1, 1:100 (Santa Cruz Biotechnology Inc.), rabbit anti-LC3, 1:200 (Abcam, Cambridge, UK). Only to detect LAMP2, fixation and permeabilization were done with methanol (10 min) and acetone (1 min). Incubation with secondary antibodies (anti-mouse TRITC-conjugated, anti-mouse Alexa-fluor 488-conjugated, anti-rabbit Alexa-fluor 488-conjugated, anti-rabbit Alexa-fluor-546-conjugated, anti-goat Alexa-fluor 488-conjugated (Thermo Fisher Scientific)) were done for 1 h in 1% bovine serum albumin in PBS at a dilution of 1:100. Finally, cover slips were washed several times with PBS and mounted with Mowiol (Sigma-Aldrich). Confocal images were acquired with a Zeiss LSM 510 laser scanning microscope (Carl Zeiss MicroImaging Inc., Iena, Germany). Magnification of the micrographs was the same for all the figures shown (63× objective). Colocalization analysis was performed with the AIS Zeiss software. Each value of the calculated colocalization index could range from 0 (no colocalization) to 1 (all pixels co-localize).

### 4.6. In Situ TG2 Activity

In situ TG2 activity was detected by a microplate assay using the TG2 substrate pentylamine-biotin (Thermo Fisher Scientific) as reported elsewhere [37], with some modifications. Briefly, fibroblasts were seeded at a density of 3000/cm^2^ in plates of 60 mm in diameter and cultured for 48 h, then treated with different amounts of p31–43, p57–68, and the control peptide p229–246 for 30 min. At the end of the incubation with peptides, cells were harvested in RIPA lysis buffer, consisting of 20 mM Tris pH 7.5, 150 mM NaCl, 10% glycerol, 0,1% SDS, 1 mM Na_3_OV_4_, 1 mM PMSF, and an inhibitor cocktail of proteases. Total lysates were centrifuged at 12,000× *g* for 10 min at 4 °C. Supernatants (25 μg) were used to coat wells of a 96 well microplate. A blocking solution consisting of 10% bovine albumin in borate buffer saline (80 mM NaCl, 100 mM H_3_BO_3,_ 20 mM Na_2_B_4_O_7_) was added to each well for 2 h. After several washes, wells were treated with horseradish peroxidase-conjugated streptavidin (Thermo Fisher Scientific) 1:3000 in borate buffer saline with 0.1% Tween 20, then color was developed by adding 3,3′,5,5′-tetramethylbenzidine. By measuring absorbances at 450 nm, we obtained an estimation of intracellular TG2 activity, in the absence and in the presence of gliadin peptides.

### 4.7. Western Blot

We performed a Western blot analysis to detect TG2 in subcellular fractions, obtained from control and celiac fibroblasts as described in Section 4.3. We used the mouse anti-TG2 antibody, clone CUB 7402, diluted 1:1000 in a 0.5% milk solution in Tris-buffered saline (50 mM Tris, pH 7.5, 150 mM NaCl). We also used anti-EGF-receptor antibodies (1:1000), anti-GAPDH antibodies (1:2000), and anti-lamin B antibodies (1:1000) as markers for membrane, cytosolic, and nuclear compartments, respectively. We also monitored modifications in TG2 expression induced by gliadin peptides. Briefly, fibroblasts were treated with 20 and 80 μg/mL of each peptide for 48 h, then cells were harvested in RIPA buffer (see Section 4.6), and 35 μg of proteins were used for Western blot analysis.

### 4.8. Real-Time PCR

To quantify TG2 mRNA levels after treatments with p31–43, we performed a real-time PCR analysis according to the protocol previously reported [24]. Fibroblasts were treated for 24 h in the presence of 20, 40, and 80 μg/mL of p31–43 before the analysis.

### 4.9. Statistics

Statistical analysis was performed where appropriate by using the Student’s *t*-test. Differences were considered to be statistically significant at *p* < 0.05.

### 4.10. Ethic Statement

The protocol for this study was approved by the Ethical Committee of the University ‘‘Federico II’’, Naples, Italy (ethical approval: C.E. n. 230/05/E1; approval date: 1 March 2006). All adult subjects provided written informed consent to use the biological material in this study. Parents or tutors provided written informed consent for subjects under 18 years of age.

## Figures and Tables

**Table 1:** Summary of differential features regarding TG2 in CD and control cultures of primary skin-derived fibroblasts.

Investigated Features	Findings in CD Versus Control Fibroblasts
Association to the membrane	More evident
Association to the cell surface	More evident
Colocalization with EEA1	More evident
Colocalization with LAMP2	Similar
Colocalization with the transferrin-receptor	Similar
Colocalization with LC3	More evident
Intracellular activity induced by p31–43	Less evident
Expression induced by p31–43	More evident

## Abbreviations

TG2	type 2 transglutaminase
CD	celiac disease
HLA	human leucocytes antigen
p31–43	α-gliadin peptide 31–43
p57–68	α-gliadin peptide 57–68
p229–246	A-gliadin peptide 229–246
LAMP2	lysosome-associated membrane protein 2
EEA1	early endosome antigen 1
LC3	microtubule-associated protein1A/1B-light chain 3
PBS	phosphate-buffered saline
